# Supplementary material for: An HIV-1/HIV-2 Chimeric Envelope Glycoprotein Generates Binding and Neutralising Antibodies against HIV-1 and HIV-2 Isolates
Source: Int J Mol Sci. 2023 May 22;24(10):9077. doi: 10.3390/ijms24109077 (PMC10219247; doi:10.3390/ijms24109077)
Supplement: Supplementary file 1 [file ijms-24-09077-s001.zip › Figure S1.pdf]

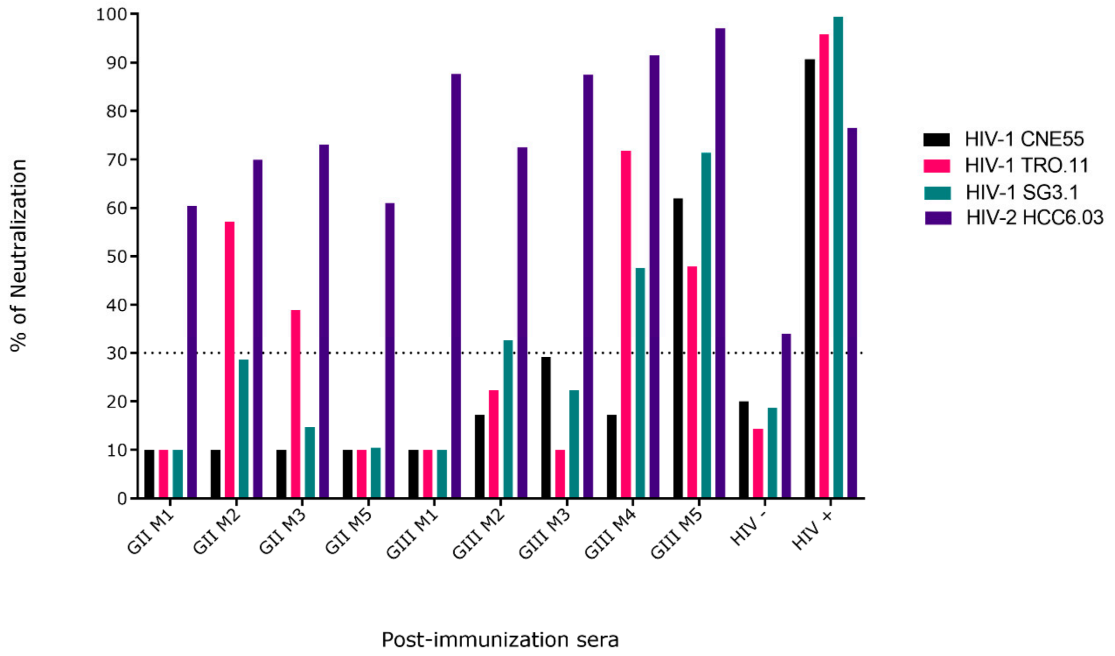

Figure S1- Neutralising activity of sera collected after the fourth boost as assessed by the percentage of neutralisation of Tier 1 (HIV-1SG3.1) and Tier 2 (HIV-1CNE55; HIV-1 TRO.11; HIV-2 HCC06.03) isolates. GII- group II mice; GIII- group III mice. HIV-, HIV-1 seronegative serum (negative control); HIV+, HIV-1 seropositive serum (positive control).
