# Supplementary material for: An HIV-1/HIV-2 Chimeric Envelope Glycoprotein Generates Binding and Neutralising Antibodies against HIV-1 and HIV-2 Isolates
Source: Int J Mol Sci. 2023 May 22;24(10):9077. doi: 10.3390/ijms24109077 (PMC10219247; doi:10.3390/ijms24109077)
Supplement: Supplementary file 1 [file ijms-24-09077-s001.zip › Figure S2.pdf]

```
HIV1HXB2   MRVKEKYQHL WRWGWRWGTM LLGMLM--IC SATEKLWVTV YYGVPVWKEA TTTLFCASDA KAYDTEVHNV WATHACVPTD  [80]
HIV1TH023 .RTQMTWPN. .K..-----T .I.LVI--.. ..SDN..... -......RD. D--....... ..QE..A... ..........  [80]
HIV2ALI   .SSRNQ---- ---------L .VTI.LASA. LVYC.QY... F....A-.N. SIP....TKN RD-------- .G.IQ.L.-.  [80]

HIV1HXB2   PNPQEVVLVN VTENFNMWKN DMVEQMHEDI ISLWDQSLKP CVKLTPLCVS LKCTDLKNDT NTNSSSGRMI ME--------  [160]
HIV1TH023 .....LH.E. .......... -.....Q..V -......... .........T .N..NANVTN VK.ITNVPN. GNI-------  [160]
HIV2ALI   NDY..IA.-. ...A.DA.DT --.TEQA..V WR.FET.I.. ........-A M..SNISTES T.SP.P.STL KPIESDPCIK  [160]

HIV1HXB2   --------KG EIKNCSFNIS TSIRGKVQKE YAFFYKLDII --PIDNDTTS YK--LTSCNT SVITQACPKV SFEPIPIHYC  [240]
HIV1TH023 --------TD .VR.....MT EL-.D.K..V H.L.....V- --..E.-SSK .R--.IN... ...K.....I ..D.......  [240]
HIV2ALI   ADNCPRGLDE .MV..R..MT GLQ.D.PKQY NETW.SK.VV CE.FNTT.NQ TRCYMNH... ....ES.D.H YWDA.RFR..  [240]

HIV1HXB2   APAGFAILKC NNKTFNGTGP -CTNVSTVQC THGIRPVVST QLLLNGSLAE EEVVIRSVNF TDNAKTIIVQ LNTSVEINCT  [320]
HIV1TH023 T.-.Y..... .D.N...... -.K.-.S... ....K.A... .......... ..II...E.L .N.......H ..K.......  [320]
HIV2ALI   ..P.Y.L.R. .DTNYS.FA. N.SK.VAAT. .RMMEMQT.. WFGF..TR.. NRTY.YWHG- -RDNR...LN KQYNLTMH.-  [320]

HIV1HXB2   RPNNNTRKRI RIQRGPGRAF VTIGKI-GNM RQAHCNISRA KWNNTLKQIA SKLREQ---- --FGNNKTII FKQSSGGDPE  [400]
HIV1TH023 ..S....TS. N.--...QV. YRT.D.I.DI .K.Y.E.NG. ...EV..KVT E..K.H---- --.-.....F ---PPS..L.  [400]
HIV2ALI   K.G.K.VVP. TLMS.----L FHSQP.NKRP ...W.WFKG- E.REAMQEVK ET.VKHPRYK GTNDT.QINF T.PGR.S.A.  [400]

HIV1HXB2   IVTHSFNCGG EFFYCNSTQL FNSTWFNSTW STEGSNNTEG SDTITLPCRI KQIINMWQKV GKAMYAPPIS GQIRCSSNIT  [480]
HIV1TH023 .M-.H...R. ..-...T.R. ..N.------ ----CMEN.T MCG.I...K. ........RA .Q........ .R.N.V....  [480]
HIV2ALI   E.-YMWT.R. .L-...M.WF L.WV------ ----E.K.GQ EQHNYA..H. ..-..T.H.. ..NV.L.R-E .ELT.-NTV.  [480]

HIV1HXB2   GLLLTRDGGN SNNESEIFRP GGGDMRDNWR SELYKYKVVK IEPLGVAPTK AKRRVVQREK RAVGIGALFL GFLGAAGSTM  [560]
HIV1TH023 .I..-....- L..TN.T... ...NIK.... .........Q .....I...R ......E... .......MIF ......-...  [560]
HIV2ALI   S.IANI.--- -TDQTIT.SA EVAEL---Y. L..GD..-.E .T.I.F.TSE RRYSSTP.N. ---.VFV.L. ...AT...MT  [560]
```

```
HIV1HXB2  GAASMTLTVQ ARQLLSGIVQ QQNNLLRAIE AQQHLLQLTV WGIKQLQARI LAVERYLKDQ QLLGIWGCSG KLICTTAVPW  [640]
HIV1TH023 ..--I..... .......... ..S....... .......... .........V ....--Y... -K..L....- -K.I......  [640]
HIV2ALI   A.--L..A-. S.T..A.... ..-Q..DMV- -K.QM.R... ..-.N....V -TI.K..... AR.NS...AF RQV.H.T..-  [640]

HIV1HXB2  NASWSNKSLE QIWNHTTWME WDREINNYTS LIHSLIEESQ NQQEKNEQEL -LELDKWASL WNWFNITNWL WYIKLFIMIV  [720]
HIV1TH023 .ST...R.F. E...NM..I. .E...S...N Q.YEILTQ.. ...DR..KD. -........- --..D..--. ....I...-.  [720]
HIV2ALI   ---.V.N..K PD.DNM..E. --QQVRYLEA N.SEQL.QA. I.....TY.. K.SW.VFTN- --.LDL.A-V K..QYGVY.I  [720]

HIV1HXB2  GGLVGLRIVF AVLSIVNRVR QGYSPL---- -SFQTHLPTP RGPDRPEGIE EEGGERDRDR SIRLVNGSLA -LIWDDLRSL  [800]
HIV1TH023 ...I....I. .......... ......---- -.L.--ITHH .E...-.R.- .....QG..K .V...S.F.. -.T.......  [800]
HIV2ALI   V.I.A....I Y.-QMLS.L. K..R.VFSSP PGYIQQIHIH KDQE------ QT.T.DVDNV GD..WPWPI. Y.HFHL.ALI  [800]

HIV1HXB2  CLFSYHRLRD LLLIVTRIVE LLGR-----R GWEALKYWWN LLQY---WSQ E---LKNSAV SLLNA--TAI AVAEGTDRVI  [880]
HIV1TH023 .......-.. FIS.AA.T.. ...HS-SKL. ...G...LG. I.-.---.G. .---..I..I .-...--... ...-W.....  [880]
HIV2ALI   G.Y.IC.DLS RFS.LQP.FQ S.Q.ALTTI. D.---RLKAA Y...GCE.I. .AFQFRIARE T.T.TWRDLW GAMQWVG.IL  [880]

HIV1HXB2  EVVQGACRAI RHIPRRIRQG LERILL [906]
HIV1TH023 .-A...W... L...-..... ...T.. [906]
HIV2ALI   A--------- --V....... A.IA.. [906]
```

Figure S2- Alignment of the envelope sequences of HIV-1 HXB2 (subtype B), HIV-1 TH023 (CRF01_AE) and HIV-2 ALI. Alignment was performed with Muscle using default parameters in MEGA X. The chimeric gp120 produced in this work was based on the TH023 and ALI strains. The HIV-2 ALI C2V3C3 fragment present in the chimera is shown in red letters.
